# Brain-wide functional connectivity patterns support general cognitive ability and mediate effects of socioeconomic status in youth

**DOI:** 10.1038/s41398-021-01704-0

**Published:** 2021-11-08

**Authors:** Chandra Sripada, Mike Angstadt, Aman Taxali, D. Angus Clark, Tristan Greathouse, Saige Rutherford, Joseph R. Dickens, Kerby Shedden, Arianna M. Gard, Luke W. Hyde, Alexander Weigard, Mary Heitzeg

**Affiliations:** 1 Department of Psychiatry, University of Michigan, Ann Arbor, MI USA; 2 Department of Statistics, University of Michigan, Ann Arbor, MI USA; 3 Department of Psychology and Neuroscience and Cognitive Neuroscience Program, University of Maryland, College Park, MD USA; 4 Department of Psychology and Survey Research Center at the Institute for Social Research, University of Michigan, Ann Arbor, MI USA

**Keywords:** Long-term memory, ADHD

## Abstract

General cognitive ability (GCA) is an individual difference dimension linked to important academic, occupational, and health-related outcomes and its development is strongly linked to differences in socioeconomic status (SES). Complex abilities of the human brain are realized through interconnections among distributed brain regions, but brain-wide connectivity patterns associated with GCA in youth, and the influence of SES on these connectivity patterns, are poorly understood. The present study examined functional connectomes from 5937 9- and 10-year-olds in the Adolescent Brain Cognitive Development (ABCD) multi-site study. Using multivariate predictive modeling methods, we identified whole-brain functional connectivity patterns linked to GCA. In leave-one-site-out cross-validation, we found these connectivity patterns exhibited strong and statistically reliable generalization at 19 out of 19 held-out sites accounting for 18.0% of the variance in GCA scores (cross-validated partial *η*^2^). GCA-related connections were remarkably dispersed across brain networks: across 120 sets of connections linking pairs of large-scale networks, significantly elevated GCA-related connectivity was found in 110 of them, and differences in levels of GCA-related connectivity across brain networks were notably modest. Consistent with prior work, socioeconomic status was a strong predictor of GCA in this sample, and we found that distributed GCA-related brain connectivity patterns significantly statistically mediated this relationship (mean proportion mediated: 15.6%, *p* < 2 × 10^−16^). These results demonstrate that socioeconomic status and GCA are related to broad and diffuse differences in functional connectivity architecture during early adolescence, potentially suggesting a mechanism through which socioeconomic status influences cognitive development.

## Introduction

In addition to specific abilities that contribute to the performance of individual cognitive tasks, there is considerable evidence for a general cognitive ability (GCA) [1, 2] that contributes to performance across a diverse range of cognitive tasks [3–7]. GCA is a fundamental dimension of individual differences and is associated with a suite of adaptive academic, occupational, health, and well-being-related outcomes [8–12]. Thus, there is great interest in understanding the neural underpinnings of GCA and the developmental mechanisms of inter-individual GCA differences.

The human brain is organized as a complex network [13, 14], with interconnections among regions implicated in diverse cognitive functions [15]. Network neuroscience [16] is beginning to shed light on how the brain’s connectivity architecture contributes to individual differences in GCA, especially using newer multivariate data-driven approaches [17, 18], but most existing studies of GCA have been in adult samples [19, 20]. During early adolescence, brain networks exhibit substantial maturation [21] and cognitive abilities rapidly improve [22]. Several studies examined connectivity patterns linked to specific aspects of cognition in youth, especially matrix reasoning [23]. In addition, our group [24] as well as other groups [25, 26] examined connectivity patterns linked to specific neurocognitive tasks or neurocognitive domains, each of which has some overlap with GCA, in earlier waves of the sample used here. But the relationship between whole-brain functional connectivity patterns and GCA, i.e., a general factor derived from bifactor or hierarchical modeling of cognitive tasks, during early adolescence has not been extensively studied, especially in large adequately powered samples.

Additionally, differences in socioeconomic status (SES) have been established as a robust predictor of GCA in childhood [27–29]. Gaps in standardized test scores between the top and bottom SES deciles are sizable [30]. Notably, they are larger in the United States than in other industrialized countries [31], and there is evidence they are growing larger over time [32], potentially reflecting rising structural inequality in the United States [33, 34]. These findings raise pressing questions about how exactly socioeconomic factors get “under the skin” to affect GCA. The potential role of SES in influencing individual differences in GCA via brain functional connectivity patterns has not previously been investigated, c.f. [26].

The present study investigates these critical questions by leveraging data from the Adolescent Brain Cognitive Development (ABCD) study, the largest youth neuroimaging study ever conducted [35, 36], with a racially and economically diverse sample recruited at 21 sites across the United States. We factor analyzed the ABCD neurocognitive task battery [37] that includes 11 task cognitive tasks, yielding a dominant general factor (“GCA”) that captured 75% of the variation in task scores [coefficient *ω* hierarchical [38]]. To characterize potentially highly distributed brain connectivity patterns related to GCA, we used data-driven multivariate predictive modeling methods [39] applied to resting-state functional connectomes. These methods learn a weighting function over the set of features (in the present case, functional connectivity maps), where the weights aggregate information across the brain and maximize the relationship between brain features and the phenotype of interest (in the present case, GCA scores). We in addition leveraged this multivariate methodology to assess the extent to which individual differences in distributed brain connectivity patterns explain the well-established relationship between SES and GCA [27–29].

## Methods

### Sample and data

The ABCD study is a multisite longitudinal study with 11,875 children between 9 and 10 years of age from 21 sites across the United States. The study conforms to the rules and procedures of each site’s Institutional Review Board, and all participants provide informed consent (parents) or assent (children). Detailed descriptions of recruitment procedures [40], assessments [41], and imaging protocols [42] are available elsewhere.

### Data acquisition, fMRI preprocessing, and connectome generation

High spatial (2.4 mm isotropic) and temporal resolution (TR = 800 ms) resting-state fMRI was acquired in four separate runs (5 min per run, 20 min total, full details are described in [43]). The entire data pipeline was run through automated scripts on the University of Michigan’s high-performance cluster and is described in detail in the Supplement, with additional detailed methods automatically generated by fRMIPrep software provided in a second fMRIPrep Supplement. Key features of the pipeline include FreeSurfer normalization, ICA-AROMA denoising, CompCor correction, use of the Gordon parcellation augmented with subcortical and cerebellar atlases, and censoring of high motion frames with a 0.5 mm framewise displacement threshold. A quality control-resting state functional connectivity plot is shown in Fig. S1.

### Inclusion/exclusion

There are 11,875 subjects in the ABCD Release 2.0.1 dataset. Exclusions were then applied based on: passing ABCD raw QC, visual inspection for data quality, sufficient resting-state data, minimum of 75 subjects at a site, and having demographic and neurocognitive data. These exclusions are described in more detail in the Supplement. This left 5937 subjects at 19 sites to enter our main predictive modeling analysis. Demographic characteristics of this sample are shown in Table 1, and additional demographic characteristics are presented in Table S1 and Table S2 in the Supplement.

**Table 1:** Demographic characteristics of subjects included in neuroimaging analysis.

*N*	5937
Age (mean (s.d.))	9.96 (0.62)
Female (%)	2991 (50.4)
*Race ethnicity (%)*	
White	3480 (58.6)
Black	728 (12.3)
Hispanic	1082 (18.2)
Asian	98 (1.7)
Other	549 (9.2)
No answer	–
*Highest parental education (%)*	
<HS diploma	199 (3.4)
Bachelor	1630 (27.5)
HS Diploma/GED	450 (7.6)
Postgraduate degree	2167 (36.5)
Some college	1590 (25.1)
No answer	3 (0.05)
*Household income (%)*	
<50 K	1389 (23.4)
≥100k	2450 (41.3)
≥50k and <100 K	1652 (27.8)
No answer	446 (7.5)

Mean framewise displacement for this sample was 0.21 mm, sd 0.09. We in addition created a low motion sample consisting of all subjects with mean framewise displacement less than 0.2 mm (*N* = 2,847). Mean framewise displacement for this sample was 0.14 mm, sd 0.03.

### GCA bifactor modeling

We used exploratory factor analysis and parallel analysis to arrive at a three-factor solution. A subsequent confirmatory bifactor model showed very good fit by conventional standards (*χ*^2^ (34) = 443.16, *p* < 0.001, RMSEA = 0.03, CFI = 0.99, TLI = 0.98, SRMR = 0.02), with the general factor capturing 75% of the variation in task scores [coefficient *ω* hierarchical [38]], and three domain-specific factors together accounting for 13% of the variation in task scores. Details, as well as a factor model (Fig. S2), are provided in the Supplement.

### Principal component regression predictive modeling

**Fig. 1:**
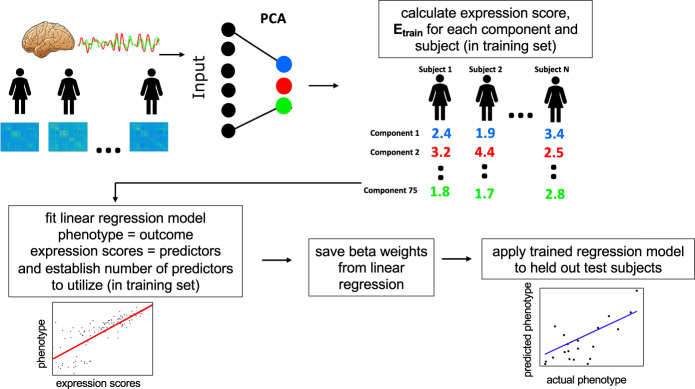
Steps of Principal Component Regression Predictive Modeling. Brain connectivity features enter data reduction yielding a smaller set of brain components. A linear regression model is fit with brain component expression scores predicting a phenotype. The components as well as betas from the linear regression are then applied out of-sample to test data to generate predicted scores and evaluate performance.

We implemented principal component regression (PCR) [44] as a multivariate predictive modeling method for identifying brain–behavior relationships [45] (see Fig. 1). We performed PCA dimensionality reduction on an *n* subject by *p* connectivity features matrix, yielding *n* principal components (i.e., directions in the feature space) that represent inter-individual differences in connectivity. Per-subject expressions scores for a subset of *k* of these connectivity components then entered multiple regression modeling to identify linear associations with phenotypes of interest (here, GCA scores). Of note, we selected *k* using five-fold cross-validation within the training data, as in our previous work [46]. We provide additional rationale for this approach in the Supplement.

### Leave-one-site-out cross-validation

To assess the accuracy and generalizability of PCR predictive models, we used leave-one-site-out cross-validation. In each fold of the cross-validation, data from one of the 19 sites served as the held-out test dataset, and data from the other 18 sites served as the training dataset. Additionally, to ensure complete separation of train and test datasets, at each fold of the cross-validation, a new PCA was performed on connectomes in the training dataset and a new factor analysis was performed on the cognitive tasks in the training dataset, and expression scores of these brain components and GCA factors were calculated for the test set. Note that by employing leave-one-site-out, members of twinships and sibships are never present in both training and test samples. We assessed the performance of PCR predictive models with cross-validated Pearson’s correlation and cross-validated partial eta squared (see Supplement for formulas).

### Accounting for covariates in a cross-validation framework

In each fold of the leave-one-site out cross-validation, PCR predictive models were trained in the train partition with the following covariates (unless explicitly stated otherwise for specific analyses): sex, race, age, age squared, mean FD, and mean FD squared. To maintain a strict separation between training and test datasets, regression coefficients for the covariates learned from the training sample were applied to the test sample to calculate effect size measures (Pearson’s correlation_cross-validated_ and partial *η*^2^_cross-validated_). This procedure is described in detail in our previous publication [47] and in the Supplement.

### Permutation testing

We assessed the significance of all cross-validation-based correlations with nonparametric permutation tests. We randomly permuted the 5937 subjects’ GCA scores 10,000 times and reran the PCR predictive modeling stream at each iteration, yielding a null distribution of correlation values. The procedure of Freedman and Lane [48] was used to account for covariates. In addition, exchangeability blocks were used to account for twin, family, and site structure and were entered into Permutation Analysis of Linear Models [49] to produce permutation orderings, as described in detail in the Supplement.

### Consensus connectome maps

To help convey overall patterns across PCR predictive models with a large number of components, we constructed “consensus” component maps. We used multi-level multiple regression modeling, with GCA scores as the outcome variables and expression scores for the components as predictors. Sex, race, age, age squared, mean FD, and mean FD squared were entered as fixed effect covariates, with family id and ABCD site entered as random effects (family nested within site). We next multiplied each connectomic component with its associated regression coefficient. We then summed across all components yielding a single map.

### SES composite score

We created an SES factor consisting of shared variance from household income, highest parental education, and an index of neighborhood disadvantage. Our neighborhood disadvantage variable follows the approach taken in [50]; see Supplement for details on these three variables. To generate SES factor scores, confirmatory factor analysis was fit using the *lavaan* package in R in which these three variables loaded on a single factor. We found the factor explained 58% of the variance, and all three variables exhibited strong loadings on the factor. Details including a factor model (Fig. S3) are provided in the Supplement. We conducted three follow-up mediation models in which each individual variable was the predictor in place of SES. These models and their results are presented in the Supplement.

### Statistical mediation analysis

We conducted the mediation analysis using a split-half approach. First, we formed 100 matched splits using the R package *MatchIt*, matching the splits on GCA, household income, highest parental education, neighborhood disadvantage, age, gender, race/ethnicity, and household marital status. Next, for each of the 100 pairs (“split *a*” and “split *b*”), we trained PCR predictive models in split *a* to predict GCA, and we applied the trained regression model to split *b* yielding expression scores for each subject that reflect each individual’s expression of the GCA-related connectivity pattern. Then in split *b*, we conducted a mediation analysis with SES scores as predictor, expression scores of brain connectivity signatures (learned from split *a*) as a mediator, and GCA scores as the outcome, and assessed statistical mediation with the mediate package in R. We entered sex, race, age, age squared, mean FD, and mean FD squared, and site ID as covariates. We performed this split-half mediation analysis 100 times, once for each pair of matched splits.

### Partial correlation connectomes

As noted above, partial correlation matrices were computed in a manner analogous to Pearson’s correlation matrices (see Methods, §2). However, in place of the Pearson’s correlation step, we computed partial correlation matrices for each run using the *ConnectivityMeasure* function from the python package *nilearn* [51]. This function computes a covariance matrix (using Ledoit-Wolf estimator), inverts the covariance matrix yielding the precision matrix, and then rescales the precision matrix yielding partial correlation connectomes.

## Results

### Brain-wide connectivity patterns are highly effective in predicting GCA scores in held-out subjects

**Fig. 2:**
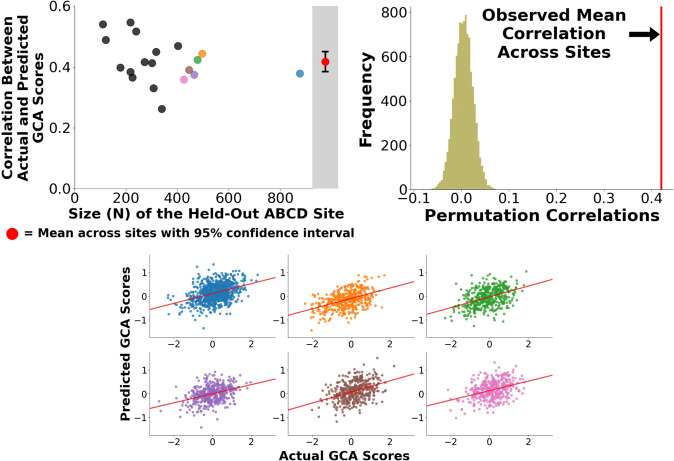
Correlations between actual GCA scores and GCA scores that are predicted based on brain connectivity. We applied multivariate predictive models to 5937 subjects at 19 sites to identify brain-wide connectivity patterns that are associated with general cognitive ability (GCA). (Upper Left Panel) In leave-one-site-out cross-validation, functional connectivity patterns associated with GCA generalized to 19 out of 19 held-out sites. (Upper Right Panel) The overall mean correlation between observed GCA scores and predicted GCA scores (predicted exclusively from brain connectivity patterns) was 0.42, *p*_PERM_ < 0.0001 (observed correlation was higher than all 10,000 correlations in the permutation distribution). (Lower Panel) Scatter plots for the six largest held-out sites (blue, orange, green, purple, brown, and pink) show highly consistent performance at individual sites.

We built and assessed predictive models for GCA using a leave-one-site-out cross-validation approach. At each fold of the cross-validation, we trained a multivariate predictive model to use individual differences in brain connectivity patterns to predict GCA. We then applied the trained model to brain connectivity data from subjects at the held-out site, yielding predictions of their GCA scores, and we repeated this sequence with each site held out once. We found that the correlation between actual versus predicted GCA scores, averaging across the 19 folds of the cross-validation, was 0.42 (Fig. 2, left panel). That is, brain connectivity patterns accounted for 18.0% of the variance in GCA scores in held-out samples of youth (cross-validated partial *η*^2^). Cross-site generalizability was remarkably consistent (Fig. 2, right panel). Correlations between predicted and actual scores were statistically significant in all 19 out of 19 held-out sites (all 19 site-specific *p*-values < 0.0001; observed correlations were higher than all 10,000 correlations in the permutation distribution). We created a consensus map that summarizes functional connectivity patterns that contributed to effective GCA prediction (Fig. 2). This map identified GCA-related connections throughout the brain, and we further examine the spatial distribution of GCA-related connectivity below. Because head motion during scanning is known to contribute to artifactual effects [52], we repeated the entire analysis in a low motion subsample (*N* = 2847). The correlation between actual versus predicted GCA scores remained strong, *r* = 0.37, indicating that head motion is unlikely to be driving our results.

### GCA-related connectivity is widely distributed throughout the brain, with minimal concentration in any individual networks

**Fig. 3:**
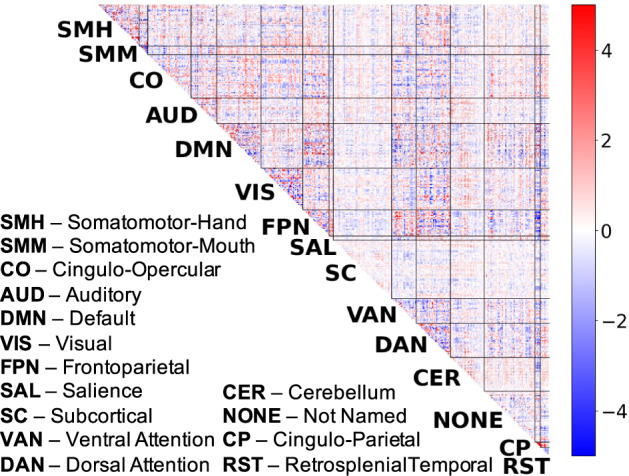
Brain connectivity patterns are predictive of GCA. We found that multivariate predictive models trained on brain functional connectivity maps were effective in predicting GCA in held-out subjects. We created a consensus map that summarizes functional connectivity patterns that contribute to effective GCA prediction.

We next examined the spatial distribution of GCA-related connectivity, focusing on the question of whether strong GCA associations are concentrated in certain networks. Visual inspection of the predictive neuro signature for GCA (Fig. 3) suggests qualitatively that GCA-related connections are highly widespread throughout the brain. We performed three additional analyses that further support this conclusion.

**Fig. 4:**
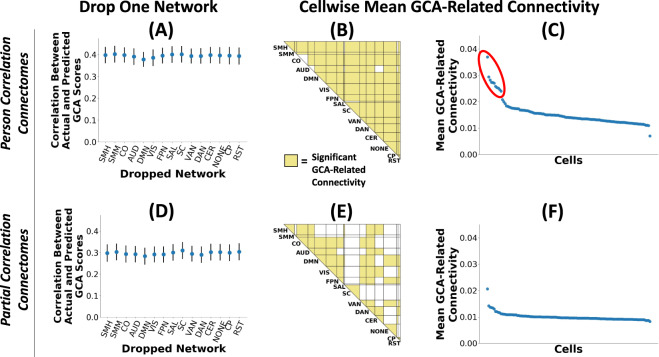
GCA-related connectivity is widely distributed throughout the brain with a minimal concentration in individual networks. We performed multiple analyses that convergently support the view that GCA-related connectivity is widespread across the connectome. **A** We repeated our multivariate predictive modeling analysis dropping one brain network each time. The relatively flat profile in the plot indicates no single network is uniquely important for predicting GCA based on brain connectivity patterns. **B** We calculated mean GCA-related connectivity for each cell (set of connections linking a pair of networks). Statistical tests revealed significantly elevated GCA connectivity at 110 of 120 network cells. **C** Plot showing mean GCA-related connectivity for each cell. These values are notably located in a narrow range. We did find somewhat elevated GCA connectivity above in 11 cells (shown in the red circle), and these 11 cells all involve either cingulo-parietal network or retrosplenial network, two small networks in posterior parietal regions. (Bottom Row: **D**–**F**). We repeated the three preceding analyses with partial correlation connectomes that allow better estimation of direct connections between regions, and the results were highly similar. SMH somatomotor-hand, SMM somatomotor-mouth, CO cingulo-opercular, AUD auditory, DMN default, VIS visual, FPN frontoparietal, SAL salience, SC subcortical, VAN ventral attention, DAN dorsal attention, CER cerebellum, NONE not named, CP cingulo-parietal, RST retrosplenial temporal.

First, we dropped one network at a time and redid the entire predictive modeling analysis stream including leave-one-site-out cross-validation. As shown in Fig. 4A, the overall prediction of GCA based on brain connectivity patterns remained similar to the original analysis without any networks dropped, and the relative flatness of the plot suggests that no single network is uniquely important for GCA prediction. Second, we quantified *mean GCA-related connectivity* for each cell: each connection’s relationship with GCA was quantified with a standardized beta (taking the absolute value), and mean GCA-related connectivity was calculated as the average of these betas for the cell. We next performed separate statistical tests at each of the 120 cells assessing whether mean GCA-related connectivity exceeds what one would expect by chance, which was established through nonparametric permutation tests. This analysis (Fig. 4B) found that a remarkable 110 of the 120 cells showed statistically significantly elevated GCA connectivity (*p* < 0.05 FDR-corrected for multiple comparisons). Third, we visualized mean GCA-related connectivity for each cell to better understand the range of variation across cells. We found (Fig. 4C) that these values were concentrated in a narrow range. We did find a cluster of elevated mean GCA connectivity (mean beta_standardized_ > 0.03) in 11 cells, which are shown in the red circle in Fig. 4. Notably, these 11 cells all involved either cingulo-parietal network or retrosplenial network, two small networks in posterior parietal regions. However, of the remaining 109 cells, 104 lie in a narrow range, with the mean standardized beta for these cells smoothly varying from 0.014 to 0.023.

All the preceding analyses were conducted with connectomes that use Pearson’s correlation as the metric of connectivity between pairs of regions, as is standard in the field [53]. A known weakness of this metric is that it captures both direct connections as well as indirect connections (e.g., X is connected to Y and Y is connected to Z, so X is indirectly connected to Z via Y), potentially exaggerating the spatial extent of GCA-related connectivity [54]. Thus, we created partial correlation connectomes that capture only direct connections between regions. However, here too, we found a flat profile in the drop-one-network plot (Fig. 4D), and a sizable number of cells distributed throughout the brain with elevated GCA-related connectivity (Fig. 4E). Moreover, when visualizing mean GCA-related connectivity for each cell (Fig. 4F), we found these values were even more tightly packed in a narrow range. With a single exception, all cells had mean standardized betas for their relationship with GCA that spanned 0.011–0.017.

Taken together, these results indicate highly broad and diffuse connectivity differences are associated with GCA, with only minimal evidence of concentration of GCA-related connectivity in specific networks.

### SES is strongly related to GCA, and brain connectivity patterns partially statistically mediated this relationship

We next examined relationships between SES and brain connectivity patterns related to GCA. We constructed a broad SES factor from three intercorrelated indicators of socioeconomic resources that spanned levels of analysis [55, 56]: household income, highest parental education, and a neighborhood disadvantage factor [based on [50], see Fig. S3]. This SES factor was found to be significantly related to GCA (beta_standardized_ = 0.32; *p* < 2 × 10^−16^) in the ABCD study, consistent with findings from numerous prior studies (21, 22). We next examined whether brain connectivity patterns statistically mediated this relationship, using a cross-validated framework. Importantly, this approach avoids bias that can arise when indirect effects of GCA-related brain connectivity patterns are quantified in the same sample in which those GCA-related connectivity patterns are themselves discovered.

We split the data into demographically matched halves 100 times, yielding 100 pairs, each with a “split *a*” and “split *b*”. At each pair, we trained a multivariate predictive model to predict GCA in split *a*. Then in split *b*, we conducted a statistical mediation analysis with SES scores as a predictor, expression scores of brain connectivity signatures (learned from split *a*) as a mediator, and GCA scores as the outcome, controlling for covariates as in previous analyses. We observed statistically significant mediation at all 100 models (*p* < 2 × 10^−16^ for all 100 models), and the mean proportion mediated was 15.4% [interquartile range: 15.1–17.7]. In the split-half mediation model whose proportion mediated value was closest to the mean across models, the total effect of SES on GCA was beta = 0.29; *p* < 2 × 10^−16^. On the indirect pathway, SES was associated with brain connectivity patterns (beta = 0.19; *p* < 2 × 10^−16^) and brain connectivity patterns were associated with GCA after controlling for SES (beta = 0.24; *p* < 2 × 10^−16^); note all betas are standardized. This indirect pathway accounted for 15.5% of the total effect of SES on GCA (95% CI: 11.8–19.9, *p* < 2 × 10^−16^).

## Discussion

Using a multivariate predictive modeling approach combined with cross-validation, this study examined joint contributions of brain connectivity patterns and SES to GCA in 5937 9- and 10-year-old participants across 19 sites in the ABCD Consortium study [37, 42]. Our results support three conclusions: (1) in early adolescence, individual differences in GCA are strongly reflected in differences in brain-wide connectivity patterns; (2) GCA-related connectivity is remarkably dispersed across the brain with minimal concentration in any networks; and (3) SES is related to GCA in part via individual variation in these neural networks. These findings highlight that diffuse neural networks that underpin GCA are related to individual differences in SES. Moreover, they invite follow-up investigation in the longitudinal ABCD dataset to better understand how socioenvironmental factors such as SES may shape connectivity patterns of the maturing brain over the course of adolescence and the years beyond, influencing important cognitive, personality, and mental health outcomes.

This is among the largest studies ever to examine links between resting-state brain connectivity patterns and GCA in youth with multivariate predictive modeling methods, c.f. [23]. There is great interest in developmental neuroscience in understanding how brain connectivity patterns contribute to psychological traits and how the brain in turn is shaped by socioenvironmental factors [57, 58]. However, the complexity and high dimensionality of the brain make tracking these etiological influences “feature by feature” challenging. Multivariate predictive modeling provides an alternative approach that generally yields much stronger brain-behavior relationships due to the aggregation of small effects distributed widely throughout the brain. Consistent with this idea, we observed a strong out-of-sample relationship between brain connectivity patterns and GCA (*r* = 0.42; partial eta squared = 18.0%), with successful generalization in 19 out of 19 held out ABCD sites. The utility of identifying brain connectivity patterns linked to psychological traits and abilities depends heavily on a consistent generalization of these patterns to new datasets collected at heterogenous sites with different subject characteristics and scanners, and this study confirms that strong generalizability is possible.

We found that GCA-related connectivity is remarkably widespread across the brain: Elevated GCA connectivity was found at 110 of 120 network cells, and these cells differed only modestly in their quantity of GCA-connectivity. Previous results with task activation maps and structural maps tended to find localization of GCA effects in frontal and parietal regions [59, 60]. Our results are instead more consistent with a recent influential study by Dubois and colleagues that found distributed functional connectivity patterns associated with GCA in an adult sample [19]. In addition, prior work by our group [24] as well as other groups [25, 26, 61] with specific neurocognitive tasks and neurocognitive domain factors also tended to find connectivity changes implicating multiple networks. The present work adds to this set of results by demonstrating both elevated as well as minimally differing levels of GCA-related connectivity across *nearly all* cells of the brain. Moreover, we showed this pattern remained even when using partial correlation connectomes that aim to estimate only direct connections between brain regions. Our results thus set the stage for further inquiry into how the highly distributed functional connectivity patterns characterized here affect the topological organization [62] of the brain and shape brain flexibility and global information sharing [63].

It is well-established that SES is associated with the development of cognitive abilities [28, 29, 56]. Relatively few studies, however, have examined the neural pathways via which SES has this effect, c.f. [64, 65]. The present study adopted a novel split-half approach that combines multivariate predictive modeling with statistical mediation analysis to examine the overlapping variance among these constructs. We showed that SES is associated with the expression of brain-wide connectivity patterns that are in turn linked to GCA. Importantly, these connectivity patterns explain ~15% of the total effect of SES on GCA. There are likely additional “proximal exposures” that might help explain why SES is associated with differences in GCA-related connectivity patterns in the brain [55]. Children from higher SES households might receive more stimulating learning environments at home and/or in schools [66, 67]. Alternatively, they might have less exposure to stressors such as financial uncertainty, violence, harsh parenting, or family conflict, some of which have previously been associated with brain connectivity changes [68]. Future research should systematically investigate the pathways by which SES produces brain connectivity changes. In addition, given evidence of partial genetic mediation of the relationship between SES and cognition [69], the association between SES and brain connectivity patterns could in part be due to shared genetic predisposition. Thus, while this study provides evidence that part of the SES-GCA relationship is explained by brain connectivity patterns, the underlying reasons why SES is associated with these brain connectivity patterns await further elucidation [55].

This study has some limitations, and care must be taken in interpreting its results. First, the study uses cross-sectional data from the baseline wave of the ABCD study. Statistical mediation results from cross-sectional data should be seen as providing only initial, tentative evidence for the proposed relationships between modeled variables [70], and stronger inferences about “mediation” and/or causal relationships require other kinds of data, such as longitudinal data or experimental manipulations [70, 71]. Second, there is a long history that must be acknowledged of research on cognitive abilities being used to stigmatize marginalized groups [72]. Thus, it bears emphasis that individual differences in cognitive abilities are not static nor should they be taken to be innate and immutable. Rather, there is sizable evidence that these differences arise from, or are highly amplified by, myriad structural inequalities in society [73–75], and these structural features of society can be targeted through individual action as well as policy interventions [76, 77].

In sum, in a large, rigorously characterized sample of youth, we identified highly distributed, brain-wide functional connectivity patterns that are linked to GCA and, moreover, that potentially help to explain connections between SES and GCA, advancing our understanding of how socio-environmental factors shape brain and behavior in youth.

## Supplementary information

Supplemental Methods and Results

Supplemental FMRIPrep Methods
