# Supplementary material for: Brain-wide functional connectivity patterns support general cognitive ability and mediate effects of socioeconomic status in youth
Source: Transl Psychiatry. 2021 Nov 8;11:571. doi: 10.1038/s41398-021-01704-0 (PMC8575890; doi:10.1038/s41398-021-01704-0)
Supplement: Supplementary file 1 — Supplemental Methods and Results [file 41398_2021_1704_MOESM1_ESM.docx]

**Supplement**

**1. FMRI Preprocessing and Connectome Generation**

Preprocessing was performed using fMRIPrep version 1.5.0 (1), a Nipype (2) based tool. Full details of the fMRIPrep analysis can be found in supplemental materials. Briefly, T1-weighted (T1w) and T2-weighted images were run through recon-all using FreeSurfer v6.0.1. T1w images were also spatially normalized nonlinearly to MNI152NLin6Asym space using ANTs 2.2.0. Each functional run was corrected for fieldmap distortions, rigidly coregistered to the T1, motion corrected, and normalized to standard space. ICA-AROMA was run to generate aggressive noise regressors. Anatomical CompCor was run and the top 5 principal components of both CSF and white matter were retained. Functional data were transformed to CIFTI space using HCP’s Connectome Workbench. All preprocessed data were visually inspected at two separate stages to ensure only high-quality data was included: After co-registration of the functional data to the structural data and after registration of the functional data to MNI template space.

Connectomes were generated for each functional run using the Gordon 333 parcel atlas (3), augmented with parcels from high-resolution subcortical (4) and cerebellar (5) atlases. Volumes exceeding a framewise displacement threshold of 0.5mm were marked to be censored. Covariates were regressed out of the time series in a single step (6), including: linear trend, 24 motion parameters (original translations/rotations + derivatives + quadratics), aCompCorr 5 CSF and 5 WM components and ICA-AROMA aggressive components, high pass filtering at 0.008Hz, and censored volumes. Next, correlation matrices were calculated for each run (Pearson’s correlations for the main analyses, partial correlations for a specific analysis described below). Each matrix was then Fisher r-to-z transformed, and then averaged across runs for each subject yielding their final connectome.


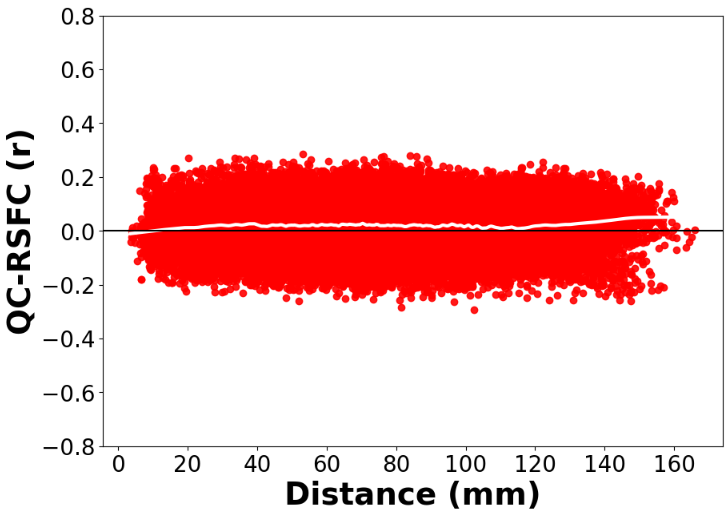


***Figure S1: Quality Control-Resting State Functional Connectivity Plot***

We used multiple procedures listed above to control the effect of head motion on brain-behavior relationships. To assess the effectiveness of these procedures, we produced a quality control resting state functional connectivity (QC-RSFC) plot (7, 8). This plot shows the relationship between mean framewise displacement and connectivity for edges binned by distance. Motion effects produce a sloped line (distance-dependent artifact), while a flat line is indicative of minimal motion-related effects. The RSFC-QC plot for our ABCD resting state data showed a flat line, providing additional evidence that our stringent motion correction strategies were effective.

**2. Bifactor Model of the ABCD Neurocognitive Battery**

Preliminary exploratory factor analyses (EFA) were first conducted (maximum likelihood estimation with oblique geomin rotation) to explore the latent structure of the 11 ABCD cognitive tasks. The scree plot, patterns of factor loadings, and a parallel analysis (with 1,000 random draws) together suggested that a three-factor solution was optimal here, where the three factors were moderately correlated. A subsequent bifactor model showed very good fit by conventional standards (χ^2^ (34)=443.16, *p*<0.001, RMSEA=0.03, CFI=0.99, TLI=0.98, SRMR=0.02), with the general factor capturing 75% of the variation in task scores [coefficient ω hierarchical (9)], and three domain-specific factors together accounting for 13% of the variation in task scores (see Figure S2). Of note, when performing cross-validation (see below), we re-estimated the bifactor model in the train dataset exclusively and applied the model to generate factor scores for test dataset subjects, thus preserving complete separation of train and test datasets.

**
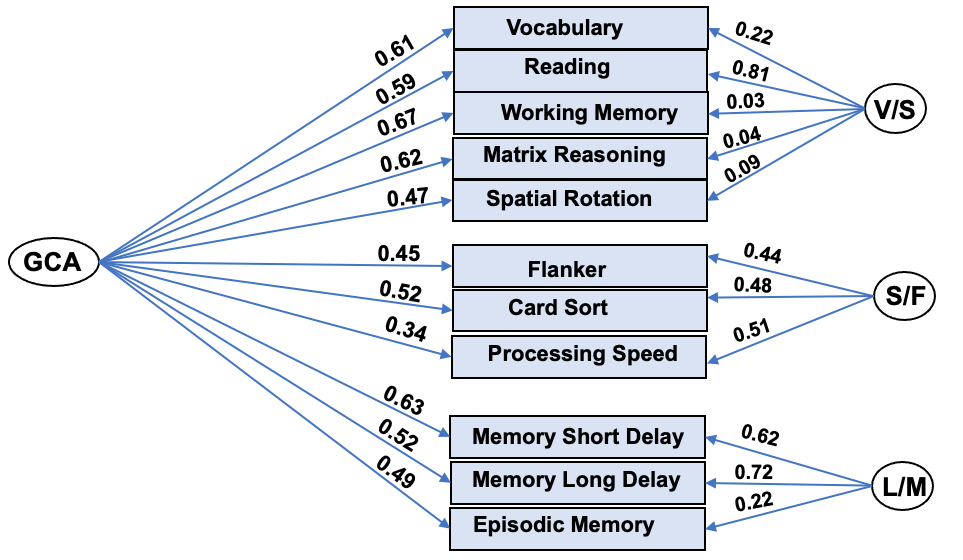
**

***Figure S2: Bifactor Model of the ABCD Neurocognitive Battery.*** *We fit a bifactor model that included a general cognitive ability factor (GCA) and three specific factors: verbal/spatial (VS), speed/flexibility (S/F), and learning memory (L/M). Path estimates reflect standardized factor loadings.*

**3. Inclusion/Exclusion**

Screening was initially done using ABCD raw QC to limit to subjects with 2 or more good runs of resting data as well as a good T1 and T2 image (QC score, protocol compliance score, and complete all =1). This resulted in 9598 subjects with 2 or more runs that entered preprocessing. Each run was subsequently visually inspected for registration as well as for warping quality, and only those subjects who still had 2 or more good runs were retained (N=8858). After connectome generation, runs were excluded if they had less than 4 minutes of uncensored data, and next subjects were retained only if they had 2 or more good runs (N=6568). Next, sites with fewer than 75 subjects were dropped. This left us with N=6449 subjects across 19 sites to enter PCA. Finally, subjects with missing values for neurocognitive scores or nuisance covariates were excluded. This left 5937 subjects to enter our main BBS predictive modeling analysis.

**4. Principal Component Regression Predictive Modeling**

We implemented principal component regression (PCR) (10) as a multivariate predictive modeling method for identifying brain behavior relationships (11) (see Figure 1). The method involves two key steps: 1) Use principal components analysis (PCA) to find a set of components that capture *inter-individual* differences in brain features; 2) Use multiple regression in a cross-validation framework to link expression scores for these components to phenotypes of interest. In previous work, we often used the more general name brain basis set (BBS) for this approach to capture commonalities with work by our group and others that use alternative methods for step 1 (e.g., independent component analysis (12, 13) or community detection (14, 15)). We chose the PCR approach for this study because our previous work showed it has high test-retest reliability (16) and predictive accuracy (17, 18), and generally performs as well or better than alternative methods such as support vector regression and ridge regression (16).

**5. Accounting for Covariates in Cross-Validation Framework**

We accounted for the effect of covariates in a cross-validation framework that maintained strict separation between training and test datasets. Regression coefficients for nuisance covariates learned from the training sample were applied to the test sample, and the variance they explain in the outcome variable was subtracted away, yielding a covariate-adjusted outcome variable, Y_adjusted_. This procedure, described in detail in our previous publication (18), allows us to estimate the contribution of brain components alone in predicting test subjects’ GCA scores, excluding the contribution of the nuisance covariates. In particular, in the test set, we calculate predicted values for the outcome variable, Y_predicted_, by multiplying brain component expression scores for the test set with their associated beta weights learned from the train dataset. Pearson’s correlation_cross-validated_ was calculated in the test set as the correlation between Y_adjusted_ and Y_predicted_. Partial η^2^_cross-validated_ was calculated as partial η^2^ for a regression model with Y_adjusted_ as the outcome and Y_predicted_ as the predictor (note that with a single predictor, partial η^2^ and η^2^ will be identical).

**6. Permutation Framework**

The distribution under chance of correlations between PCR regression predictions of neurocognitive scores and observed neurocognitive scores was generated by randomly permuting the 5,937 subjects’ neurocognitive scores 10,000 times. At each iteration, we performed the leave-one-site out cross validation procedure described in the main manuscript, including refitting PCR regression models at each fold of the cross-validation. We then recalculated the average correlation across folds between predicted versus actual neurocognitive scores. The average correlation across folds that was actually observed was located in this null distribution in terms of rank, and statistical significance was set as this rank value divided by 10,000.

Since the PCR regression models fit at each iteration of the permutation test included covariates, the procedure of Freedman and Lane (19) was followed. Since subjects were nested within families and site, exchangeability blocks (20) were generated and entered into FSL’s Permutation Analysis of Linear Models (PALM) tool (<https://fsl.fmrib.ox.ac.uk/fsl/fslwiki/PALM>) to generate 10,000 permutation orderings. At each iteration, we first fit the outcome variables (i.e., GCA) to a model containing only nuisance predictors. We calculate the residuals from this model, permute them within exchangeability blocks, then add the unpermuted estimated effect of nuisance predictors back to the permuted residuals. This new outcome variable, made up of permuted residuals and unpermuted nuisance effects, is then fit to the regression models, and then a test statistic is generated (correlation between actual and predicted GCA scores in this case). This sequence is then repeated 10,000 times in total to generate the permutation null distribution of the test statistic.

**7. SES Factor Model**


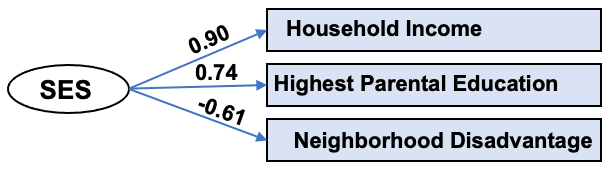


***Figure S3: Factor Model of Socioeconomic Status.*** *Path estimates reflect standardized factor loadings.*

Household Income covered all sources of income for family members, including wages, benefits, child support payments, and others. It was assessed in bins as follows: 1 <5,000, 2 5,000 - 11,999, 3 12,000 - 15,999, 4 16,000 - 24,999, 5 25,000 - 34,999, 6 35,000 - 49,999, 7 50,000 - 74,999, 8 75,000 - 99,999, 9 100,000 - 199,999, 10 More than 200,000, and we assigned each subject the natural log of the midpoint for their bin. Highest Parental Educational was the highest educational achievement by either parent or caregiver. Neighborhood Disadvantage was constructed according to the procedure of Taylor et al. (21). In brief, participant’s primary home address was used to generate Area Deprivation Index (ADI) values, which were factor analyzed and used to create an aggregate measure. Higher scores on the factor indicate greater neighborhood disadvantage including higher percent of families living in poverty, increased unemployment, and lower levels of educational attainment at the neighborhood level, see (21) for details.

**8. Mediation Models for Individual Variables in the SES Factor**

In addition to the main mediation model in which the predictor was SES scores, the mediator was brain connectivity expression scores, and the outcome was GCA scores, we conducted three additional mediation models. In each model, one component of SES—either household income, highest parental education, or neighborhood disadvantage—was used as the predictor with the mediator and outcome unchanged, and the other two components of SES were entered as covariates of no interest. We found that for mediation models for household income and highest parental education, proportion mediated remained highly statistically significant, while for the neighborhood disadvantage variable, proportion mediated was no longer significant. For the income model, the indirect effect, though highly significant, had a trivial effect size (average mediated effect in standardized units: 0.0068). For the highest parental education model, however, the size of the indirect effect was only modestly smaller than for the SES variable, and represented 15.9% of the total effect. Thus, there is evidence that over and above highest parental education’s shared variance with household income and neighborhood disadvantage, the unique variance of this variable in predicting GCA is also partially statistically mediated by GCA-related brain functional connectivity patterns.

**9. Cell-Wise Mean GCA-Related Connectivity**

We fit multiple regression models at each connection in which GCA was the outcome variable and the edge connectivity was the predictor, controlling for sex, race, age, mean FD, and mean FD squared, and we retained the absolute value of the standardized betas for edge connectivity from these models. Mean cell-wise GCA connectivity was calculated as the mean of these standardized betas for all connections in a cell. To assess whether mean cell-wise GCA connectivity was different than zero, we conducted permutation tests separately for each cell. GCA scores were shuffled 10,000 times, mean cell-wise GCA connectivity was recalculated at each iteration, and a *p*-value was obtained for each cell by locating the observed mean cell-wise GCA connectivity value in this null distribution, and then these p-values were corrected for 120 multiple comparisons with the false discovery rate procedure (22) with alpha set at *p*<0.05. As above (§8), the procedure of Freedman and Lane (19) was used to account for covariates, and exchangeability blocks were used to account for twin, family, and site structure.

|  | Included in Predictive Model | Not Included in Predictive Model |
| --- | --- | --- |
| N | 5937 | 5938 |
| Age (mean (s.d.)) | 9.96 (0.62) | 9.86 (0.62) |
| Female (%) | 2991 (50.4) | 2690 (45.3) |
| Race Ethnicity (%) |  |  |
| White | 3480 (58.6) | 2698 (45.4) |
| Black | 728 (12.3) | 1052 (17.7) |
| Hispanic | 1082 (18.2) | 1327 (22.3) |
| Asian | 98 (1.7) | 175 (3.0) |
| Other | 549 (9.2) | 686 (11.6) |
| No answer | -- | -- |
| Highest Parental Education (%) |  |  |
| < HS Diploma | 199 (3.4) | 393 (6.6) |
| Bachelor | 1630 (27.5) | 1384 (23.3) |
| HS Diploma/GED | 450 (7.6) | 681 (11.5) |
| Post Graduate Degree | 2167 (36.5) | 1876 (31.6) |
| Some College | 1590 (25.1) | 1590 (26.8) |
| No answer | 3 (0.05) | 14 (2.4) |
| Household Marital Status – Married (%) | 4254 (71.7) | 3736 (62.9) |
| Household Income (%) |  |  |
| <50K | 1389 (23.4) | 1833 (30.9) |
| >=100k | 2450 (41.3) | 2115 (35.6) |
| >=50k & <100K | 1652 (27.8) | 1418 (23.9) |
| No answer | 446 (7.5) | 572 (9.6) |

***Table S1: Demographic Characteristics of Included Versus Excluded Subjects***

| **ABCD Site ID** | **Number of Subjects** | **% Female** | **%**  **White** | **%**  **Black** | **% Hispanic** | **%**  **Asian** | **%**  **Other** | **Mean Age (std dev)** |
| --- | --- | --- | --- | --- | --- | --- | --- | --- |
| site01 | 91 | 56.04 | 18.68 | 4.4 | 52.75 | 10.99 | 13.19 | 9.89 (0.62) |
| site02 | 434 | 48.62 | 65.21 | 1.15 | 21.43 | 0.46 | 11.75 | 10.11 (0.63) |
| site03 | 444 | 50.45 | 8.33 | 16.67 | 70.5 | 0.68 | 3.83 | 9.88 (0.63) |
| site04 | 296 | 54.05 | 48.99 | 9.46 | 17.57 | 1.01 | 22.97 | 9.78 (0.65) |
| site05 | 250 | 53.60 | 62.00 | 23.20 | 3.20 | 1.20 | 10.40 | 9.93 (0.63) |
| site06 | 400 | 53.25 | 69.75 | 2.75 | 15 | 3 | 9.5 | 9.98 (0.58) |
| site07 | 204 | 50.49 | 57.84 | 17.65 | 12.75 | 2.45 | 9.31 | 9.87 (0.63) |
| site09 | 254 | 53.54 | 30.71 | 8.66 | 38.19 | 8.66 | 13.78 | 9.99 (0.61) |
| site10 | 167 | 50.9 | 29.34 | 3.59 | 54.49 | 1.2 | 11.38 | 9.91 (0.63) |
| site11 | 290 | 52.07 | 61.72 | 17.24 | 9.66 | 0.69 | 10.69 | 9.87 (0.65) |
| site12 | 117 | 47.86 | 48.72 | 27.35 | 8.55 | 1.71 | 13.68 | 9.85 (0.59) |
| site13 | 205 | 52.2 | 62.44 | 15.12 | 9.76 | 3.41 | 9.27 | 9.86 (0.60) |
| site14 | 437 | 48.51 | 80.78 | 4.81 | 5.95 | 0.69 | 7.78 | 10.24 (0.56) |
| site15 | 214 | 48.13 | 38.32 | 49.07 | 3.74 | 0 | 8.88 | 9.96 (0.60) |
| site16 | 836 | 44.98 | 81.58 | 1.08 | 11.24 | 1.08 | 5.02 | 9.90 (0.66) |
| site17 | 305 | 51.48 | 87.21 | 1.64 | 3.28 | 1.31 | 6.56 | 9.84 (0.62) |
| site19 | 204 | 54.9 | 57.35 | 23.53 | 7.35 | 0.98 | 10.78 | 10.13 (0.55) |
| site20 | 441 | 54.2 | 61.22 | 28.8 | 2.27 | 0.23 | 7.48 | 10.09 (0.49) |
| site21 | 348 | 46.26 | 53.16 | 16.09 | 20.98 | 1.72 | 8.05 | 9.95 (0.63) |
| **Total** | **5938** | **50.37** | **58.61** | **12.26** | **18.22** | **1.65** | **9.26** | **9.96 (0.62)** |

***Table S2:*** ***Demographic Characteristics of Included Subjects by Site***

**References**

1. O. Esteban, *et al.*, fMRIPrep: a robust preprocessing pipeline for functional MRI. *Nature methods* **16**, 111 (2019).

2. K. Gorgolewski, *et al.*, Nipype: a flexible, lightweight and extensible neuroimaging data processing framework in python. *Frontiers in neuroinformatics* **5**, 13 (2011).

3. E. M. Gordon, *et al.*, Generation and Evaluation of a Cortical Area Parcellation from Resting-State Correlations. *Cereb. Cortex* **26**, 288–303 (2016).

4. Y. Tian, D. S. Margulies, M. Breakspear, A. Zalesky, Hierarchical organization of the human subcortex unveiled with functional connectivity gradients. *bioRxiv* (2020).

5. J. Diedrichsen, *et al.*, Imaging the deep cerebellar nuclei: a probabilistic atlas and normalization procedure. *Neuroimage* **54**, 1786–1794 (2011).

6. M. A. Lindquist, S. Geuter, T. D. Wager, B. S. Caffo, Modular preprocessing pipelines can reintroduce artifacts into fMRI data. *Human brain mapping* **40**, 2358–2376 (2019).

7. J. D. Power, *et al.*, Methods to detect, characterize, and remove motion artifact in resting state fMRI. *Neuroimage* **84**, 320–341 (2014).

8. J. D. Power, B. L. Schlaggar, S. E. Petersen, Recent progress and outstanding issues in motion correction in resting state fMRI. *Neuroimage* **105**, 536–551 (2015).

9. R. E. Zinbarg, W. Revelle, I. Yovel, W. Li, Cronbach’s α, Revelle’s β, and McDonald’s ω H: Their relations with each other and two alternative conceptualizations of reliability. *psychometrika* **70**, 123–133 (2005).

10. I. T. Jolliffe, A note on the use of principal components in regression. *Applied Statistics* **31**, 300–303 (1982).

11. C. Sripada, *et al.*, Basic Units of Inter-Individual Variation in Resting State Connectomes. *Scientific Reports* **9**, 1900 (2019).

12. D. Kessler, M. Angstadt, C. Sripada, Brain Network Growth Charting and the Identification of Attention Impairment in Youth. *JAMA Psychiatry* **73**, 481–489 (2016).

13. D. Kessler, M. Angstadt, R. C. Welsh, C. Sripada, Modality-spanning deficits in attention-deficit/hyperactivity disorder in functional networks, gray matter, and white matter. *Journal of Neuroscience* **34**, 16555–16566 (2014).

14. J. Arroyo, *et al.*, Inference for multiple heterogeneous networks with a common invariant subspace. *arXiv preprint arXiv:1906.10026* (2019).

15. K. Levin, A. Lodhia, E. Levina, Recovering low-rank structure from multiple networks with unknown edge distributions. *arXiv preprint arXiv:1906.07265* (2019).

16. A. Taxali, M. Angstadt, S. Rutherford, C. Sripada, Boost in Test-Retest Reliability in Resting State fMRI with Predictive Modeling. *Cerebral Cortex* **31**, 2822–2833 (2021).

17. C. Sripada, *et al.*, Prediction of Neurocognition in Youth From Resting State fMRI. *Molecular Psychiatry* **25**, 3413–3421 (2020).

18. C. Sripada, M. Angstadt, S. Rutherford, A. Taxali, K. Shedden, Toward a “treadmill test” for cognition: Improved prediction of general cognitive ability from the task activated brain. *Human Brain Mapping* (2020).

19. D. Freedman, D. Lane, A Nonstochastic Interpretation of Reported Significance Levels. *Journal of Business & Economic Statistics* **1**, 292–298 (1983).

20. A. M. Winkler, G. R. Ridgway, M. A. Webster, S. M. Smith, T. E. Nichols, Permutation inference for the general linear model. *Neuroimage* **92**, 381–397 (2014).

21. R. L. Taylor, S. R. Cooper, J. J. Jackson, D. M. Barch, Assessment of neighborhood poverty, cognitive function, and prefrontal and hippocampal volumes in children. *JAMA network open* **3**, e2023774–e2023774 (2020).

22. Y. Benjamini, Y. Hochberg, Controlling the False Discovery Rate: A Practical and Powerful Approach to Multiple Testing. *Journal of the Royal Statistical Society. Series B (Methodological)* **57**, 289–300 (1995).
